# Individualized quality of life, standardized quality of life, and distress in patients undergoing a phase I trial of the novel therapeutic Reolysin (reovirus)

**DOI:** 10.1186/1477-7525-3-7

**Published:** 2005-01-27

**Authors:** Linda E Carlson, Barry D Bultz, Donald G Morris

**Affiliations:** 1 Department of Psychosocial Resources, Tom Baker Cancer Centre Holy Cross Site, 2202 Second St. SW. Calgary, Alberta, Canada; 2 Department of Oncology, University of Calgary, Alberta, Canada; 3 Department of Psychology, University of Calgary, Alberta, Canada; 4 Department of Psychiatry, University of Calgary, Alberta, Canada; 5 Department of Medicine, University of Calgary, Alberta, Canada

**Keywords:** Quality of life, Phase I trials, advanced cancer, depression, spirituality

## Abstract

**Background:**

The purpose of this study was to evaluate the individualized and standardized quality of life (QL) and psychological distress of patients participating in a Phase I trial of the novel therapeutic reovirus (Reolysin).

**Methods:**

16 patients with incurable metastatic cancer were interviewed prior to being accepted into the phase I trial with a semi-structured expectations interview, the Schedule for the Evaluation of Individual Quality of Life – Direct Weighting (SEIQoL-DW), the European Organization for Research and Treatment of Cancer Quality of Life Questionnaire (EORTC QLQ-C30), the Brief Symptom Inventory (BSI), the Beck Depression Inventory (BDI), and the Spiritual Health Inventory (SHI).

**Results:**

Patients were able to complete all measures. They felt hopeful and excited about the trial, with about two thirds hoping for disease regression and one third hoping for a cure. The most commonly spontaneously nominated areas of QL were family relationships, activities and friends, and the overall SEIQoL mean index score was 69. Health was nominated by only 38% of the sample. Scores on the SEIQoL were correlated with global QL on the EORTC QLQ C-30. Scores on the BDI and BSI were lower than reported for similar populations, and on the SHI scores were similar to other samples. Global QL on the EORTC QLQ C-30 and depression scores were associated with time to death in the nine patients who had died at the time of writing.

**Conclusions:**

Individualized QL is easy to assess in seriously ill cancer patients, provides useful information relative to each individual, and is related to standard QL measures. Repeated assessment of individualized QL of patients in Phase I trials would be a useful addition to the research.

## Background

As health care professionals begin to understand the importance of quality of life (QL) and emotional and social well-being in the treatment and progression of cancer, it has become standard practice, and in fact has been described as a bioethical imperative, to include QL assessment in all oncology clinical trials [1-7]. This is particularly the case in Phase I trials for novel therapeutics, where in many cases the patients admitted have exhausted other treatment options and likely face death within months under best supportive care [8]. A Phase I trial is the first test of a new therapeutic in humans and aims to establish a maximum tolerated dose, to evaluate dose limiting toxicity, and to examine the drug's pharmacology. There is generally little chance of clinical disease response and relatively high potential risk of toxicity in this type of trial. For patients in this situation, quality of life remains perhaps the most important variable to consider in the evaluation of the new treatment [9,10].

We report here on the initial QL of patients enrolled in the Reolysin Phase I trial, the first clinical usage of the reovirus in humans with cancer. The reovirus as a possible treatment for cancer attracted a great deal of media attention when the journal Science, in 1998, published very encouraging results showing tumor regression in animal models [11]. Further work has established the reovirus as a potential therapeutic in the treatment of brain, colorectal, ovarian and breast cancer cell lines [12,13]. A small sample of patients with different types of primary cancers that had exhausted other treatment options, all of whom had subcutaneous tumors that were easily palpable and injectable with Reolysin, were enrolled in the current trial.

Due to the nature of the population under study, it seemed especially important to use instrumentation that was flexible enough to allow these very ill patients to identify what was important to each of them as individuals, as well as to collect data from standardized instruments that would allow direct comparisons with other patient populations. Thus, a battery of tests to measure both individualized and standardized QL, distress levels, and spirituality was selected. Important aspects of QL were measured, including physical, psychological, emotional, social, and spiritual functioning, with emphasis on mood states and psychopathology. Expectations and hopes regarding participation in the trial were also elicited during a semi-structured interview.

The specific instruments used included an interview-based subjective measure of QL, the Schedule for the Evaluation of Individual Quality of Life – Direct Weighting (SEIQoL – DW)[14]. The SEIQoL-DW is a relatively new measure and unique in QL measurement in that it elicits from patients their own self-generated list of the "five most important domains of QL" for them, rather than asking about pre-set areas. After patients identify their most important domains, they rate how well each domain is for them currently, and how important overall in their lives each domain is. This instrument and its predecessor, the SEIQoL [15], have been used in oncology in several published studies [16-18]. The SEIQoL differs from the SEIQoL-DW in that a procedure called judgement analysis is used in the SEIQoL to arrive at the relative importance of each of the domains for each patient. This process is much more time consuming, abstract and complicated than the direct weighting procedure used in the SEIQoL-DW. While the SEIQoL procedure has been deemed overly burdensome for some patients with both early and advanced cancer [16,17], primarily due to the judgement analysis portion of the procedure, the SEIQoL-DW has been found to be acceptable and practical to use in a validation study of patients on Phase I clinical trials [18]. Another study of the SEIQoL-DW found that patients with advanced cancer were good judges of their own QL, and able to complete the interview with little difficulty [17].

In past studies, the most important areas of QL that patients on Phase I clinical trials identified were equally health and family [18]. However, in a sample of advanced cancer patients family concerns were consistently identified as more important than health issues [17]. The patient population in the Campbell & White study [18] was not defined except by their participation in Phase I trials, whereas the Waldron & O'Boyle sample [17] all had advanced incurable cancer. It may be the case that as illness severity progresses, concerns are directed toward domains that offer more hope. Other studies that have used the SEIQoL found family, health and finances to be the top three areas in men with early stage prostate cancer [16], and similarly family, health, marriage and leisure/hobbies were most important to a group of cardiac patients [19]. Thus, although there does seem to be some consistency in the areas nominated by diverse patient groups, differences in the relative importance of the areas are commonly found.

The other QL questionnaire used in this study is the very widely used European Organization of Research and Treatment of Cancer (EORTC) Quality of life Questionnaire (QLQ C-30), a 30 item standardized self-administered questionnaire that taps into important domains of QL, including physical, psychological, emotional and social functioning. This was included to allow comparisons with a well-known and validated standardized quality of life questionnaire with set domains and subscale scores. Another study at our Centre directly compared the SEIQoL to the EORTC QLQ C-30 in a sample of early stage prostate cancer patients [16]. The authors compared the domains nominated by the patients to those included on the EORTC QLQ C-30, and concluded that although there was substantial overlap on some items, many items identified by patients were not included on the standardized questionnaire.

The area of spirituality was also of interest in this population of very ill people, as issues of death and dying are often accompanied by questioning in the realm of spirituality. The Spiritual Health Inventory [20] was used for this purpose, as it measures self-acceptance, relationships with others, and hope, which may be an important factor as patients participate in the trial. In terms of depression, anxiety and other psychiatric symptoms that are frequent in cancer patients, the Brief Symptom Inventory (BSI)[21] was used to broadly assess many areas of psychopathology, and the Beck Depression Inventory (BDI)[22] to focus in more detail on depressive symptomatology. Both of these instruments are widely used in the oncology literature and thus there are many published reports with values that can be used for comparison purposes.

Previous work with patients in Phase I trials has found that patients' expectations going into trials are generally more optimistic than oncologists'. Patients with incurable malignancy in a Phase I trial estimated a greater potential for benefit from the experimental therapy than did oncologists [10]. They also estimated that the experimental therapy had less potential for toxicity than the standard treatment. The oncologists estimated the potential for toxicity on both treatments to be about equal, and lower, than did patients. Patients in Phase I trials for new drugs, when asked why they had agreed to try the new treatment, cited the potential for helping their disease to be the number one reason for participation [9]. Indeed, despite cautious words from the medical staff, it is not surprising that patients with a disease resistant to all other treatments might hope for at least a slowing of their disease progression with an experimental treatment. Thus, the preliminary QL of such patients, as measured in the current study, may be inflated by these hopes. Other studies looking at the effects of participation in Phase I trials on QL have found either no detrimental effects of participation [8], or enhancement of QL over the course of the trial compared to a control group that received supportive care [23].

The purpose of the current study was to investigate individualized QL in a group of patients with metastatic incurable cancer participating in a Phase I trial of a highly media lauded new therapeutic, and investigate their expectations regarding the trial. Areas of importance and SEIQoL Index scores for these patients will be compared with those of patients in other studies, and compared to their own scores on the standardized QL and psychological measures assessed. Relationships between the different measures will also be explored.

## Methods

### Subjects

Patients were recruited as specified in the protocol: "A Phase I Clinical Trial to Evaluate Dose Limiting Toxicity and Maximum Tolerated Dose of Intralesional Administration of REOLYSIN for the Treatment of Histologically Confirmed Malignancies". All patients had histologically confirmed evaluable palpable tumors of any histological type that had failed to improve on existing standard therapy. The injectable lesion was required to be between 1 and 10 cm^2^, and accessible and measurable for delivery of an intralesional injection. Patients were required to have a life expectancy of at least 12 weeks and a ECOG performance status of ≤ 3 and must not have received active cancer treatment for least 21 days prior to entrance onto the trial. Adequate organ reserve in terms of bone marrow, hepatic, renal and cardiac functions was required. Patients on immunosuppressive therapy or alternative/complimentary/unproven systemic or local therapies were ineligible.

### Procedures

After patients had been referred to the above mentioned trial, but prior to being definitively accepted (pending complete assessment of inclusion/exclusion criteria), patients met with a psychologist (LC) for the assessment protocol. At that time they were interviewed concerning their expectations about their health, both without any further conventional treatment and with the potential experimental treatment. They then completed the interview-based individualized QL interview, followed by the quantitative questionnaires as detailed below. All 16 patients followed the same procedures.

### Instruments

#### Demographics Form

Demographic information including age, education, marital status, occupation and current employment status was obtained on a form created for this study. Medical history including type of illness, dates of first diagnosis and subsequent relapses and site of metastases were collected, and later verified from patient charts.

#### Expectations Interview

Patients were asked three questions in a semi-structured interview: How do you feel about potentially being a part of this trial? How do you see your disease progressing without any further conventional treatment? Once on the trial, how do you see your disease progressing? Short answers to these questions were recorded verbatim at the time of the interview.

### Quality of Life

#### Schedule for the Evaluation of Individual Quality of Life – Direct Weighting (SEIQoL – DW) [14]

This schedule takes the form of a semi-structured interview, in which the investigator first describes quality of life as an individually defined construct, then elicits from the patient their own five most important domains of QL, rather than asking them about pre-set areas. Patients are asked to think of what areas of life determine their own happiness, or quality of life. After patients identify their most important domains ("cues"), they rate the quality of each domain currently in their lives by drawing a bar graph on a 100 mm scale from worst possible to best possible. This is called the "level" of the cue. Finally, they rate how important each domain is overall in their lives using a direct weighting disk. This disk consists of five overlapping different colored laminated disks that can be rotated around a central point to form a pie chart. Each piece represents one of the five chosen domains. The patient manipulates the disk until the proportion of each piece making up the pie represents the relative importance of that domain in their lives ("weight"). The weight value of each cue is calculated by determining the percentage of the overall pie that each piece covers by reading off a larger backing disk that is labeled with a 0–100 scale around the pie. Then by multiplying the level of each domain by its weight and summing the product for all five items, a summary score representing overall subjective quality of life can be calculated. This is called the SEIQoL Index Score. More detailed descriptions of the procedures are available in other publications [14,18,24].

#### The European Organization for Research and Treatment of Cancer (EORTC) QLQ-C30 Quality of Life Questionnaire [25]

This 30-item questionnaire includes five functional domains of quality of life: physical function (5 items), emotional function (4 items), cognitive function (2 items), social function (2 items) and role function (2 items). There are also several symptom scales: fatigue (3 items), pain (2 items), nausea and vomiting (2 items), and one item each for dyspnea, sleep disturbance, appetite, constipation, diarrhea and financial difficulties. Finally, two items assess global quality of life. The questionnaire shows high internal consistency, and overall reliability and validity of the survey has been demonstrated in international clinical trials with cancer patients of heterogeneous diagnoses including lung cancer [26].

### Distress

#### Beck Depression Inventory (BDI) [22]

This 21-item questionnaire gives a global score on depressive symptoms, and norms are available for many different populations, including cancer patients. Higher scores represent more depressive symptoms.

#### Brief Symptom Inventory (BSI) [21]

A general mental health measure of 58 questions which provides scores on nine dimensions of psychopathology or psychological distress: somatization, obsessive-compulsive, interpersonal sensitivity, depression, anxiety, hostility, phobic anxiety, paranoid ideation, and psychoticism. Three global scores can be calculated: the Global Severity Index (GSI), the Positive Symptom Total (PST) and the Positive Symptom Distress Index (PSDI). The GSI was used as the global score in this study.

### Spirituality

#### Spiritual Health Inventory (SHI) [20]

This instruments defines spiritual health as the capacity to transcend oneself and meet three basic needs; the need for self-acceptance, the need for relationships with others and/or a supreme being, and the need for hope. These three factors accounted for 71% of the variance in validity studies. A single total score is calculated by summing all items. The possible range of scores is 31–155, with higher scores indicating higher levels of spiritual health. The 31-item questionnaire takes very little time to complete.

## Results

### Subjects

Demographic characteristics and disease variables of participants are presented in Table 1. Patient #10 was registered in the trial but too ill to complete any of the questionnaires or the interview. Therefore no data for this patient is included in the study. The remaining 16 patients who provided data all had metastatic disease that was considered incurable, 6 men and 10 women. The largest patient group consisted of five women who had metastatic breast cancer, followed by three patients with malignant melanoma. Patients ranged in age from 32 to almost 76 years old, with a median age of 53 years. They had been diagnosed with cancer for a median of 3.3 years (range 0.5–26.9 years) before entrance to the study. They had on average 16 years of education (range 12–25), and therefore represented a highly educated group. At the time of analysis, nine of the patients had died, at a median of 136 days from the time of the interview (range 21–664 days). The remaining seven were still alive, a median of 242 days from the time of the interview (range 207–709 days).

**Table 1:** Demographic and Disease Characteristics

Patient Number	Gender	Age (Years)	Cancer diagnosis	Years since first diagnosis	Location of metastases	Days from interview to death
1	Female	45	Mucoepidermoid carcinoma (head and neck)	14.7	Lymph nodes	28
2	Male	50	Squamous carcinoma (head and neck)	0.7	Lymph nodes	Alive-709
3	Female	54	Anaplastic thyroid carconoma	10.8	Lungs/liver/bone	21
4	Female	47	Malignant Melanoma	12.7	Eye/breast/liver/chest wall/lung	664
5	Female	47	Metastatic Breast carcinoma	6.6	Chest wall/bone	241
6	Female	60	Metastatic Breast carcinoma	2.7	Chest wall /lungs /retroperitoneum	Alive-529
7	Male	32	Soft tissue sarcoma	0.5	Right lower extremity	96
8	Male	56	Neuroendocrine islet cell tumor	2.4	Liver/face/neck/ Scalp	44
9	Female	56	Metastatic Breast carcinoma	2.7	Chest wall/ left supraclavicular skin/ intrabdomen	142
11	Male	42	Malignant melanoma	6.4	Liver/lung/spleen	Alive-368
12	Male	64	Klatskin's tumor	1.4	Abdomen	171
13	Female	76	Metastatic Breast carcinoma	26.9	Lung/bone/liver	136
14	Female	55	Malignant melanoma	1.8	Axilla /lung /liver	Alive-242
15	Female	48	Metastatic breast carcinoma	2.8	Neck /Chest Wall /Brain	Alive-242
16	Female	46	Soft tissue sarcoma	3.9	Lung/skin/breast/ retroperitoneal	Alive-227
17	Male	70	Squamous carcinoma (head and neck)	9.7	Head/neck	Alive-207

### Expectations Interview

Interviews were conducted with all of the 16 patients. To the question "How do you see your disease progressing without further conventional treatment?", nine of the patients indicated they felt it would get worse and they would eventually die of their disease. This was stated in different ways: Nothing else left...getting gradually worse...terminal – could be months, could be years...wouldn't go very well. The other seven patients offered more hopeful or neutral prognoses: don't think about it – stay positive...still hopeful and optimistic...other things available still...would still take chemo, hope it would work...wouldn't ever give up on hope...faith...not sure, unknown. When asked how they felt about being in the trial, most patients indicated feeling excited, fortunate, grateful and hopeful. One indicated that they felt scared as well as hopeful, not knowing what to expect, and one said they felt like a guinea pig. To the question "Once on the trial, how do you see your disease progressing?", ten of the patients mentioned hoping for the tumor to shrink, for a remission, or for some extension of life. Five patients mentioned hoping for a cure, to be cancer free. One patient just mentioned hoping to help others, and four others said that although they were hoping for personal benefit, if it didn't help them it might help others in the future. In general, patients were hopeful yet philosophical about the trial. The 54-year old woman with melanoma captured these sentiments with her comments: It feels hopeful. Maybe it won't help me – I won't be disappointed. It might help others down the road. It's on the frontier – exciting. If it works it's a bonus. I don't totally expect anything. It may extend life. Just day by day carry on.

### EORTC QLQ C-30

Quality of life scores on the EORTC QLQ C-30 are presented in Table 2. All 16 patients completed the questionnaire. On the functional scales, where higher scores indicated better functioning, scores ranged from a low of 55 on social functioning, to a high of 76 for cognitive functioning, on a scale of 1–100. The overall global QL rating was 57. On the symptom scales, where higher scores indicate more symptomatology, scores ranged from a low of 14 (nausea and diarrhea) to a high of 42 on pain and fatigue. The next most prevalent symptoms were sleep problems and appetite loss.

**Table 2:** EORTC Scores

Functional Scales (Higher scores = higher function)	Mean	SD
Physical Function	63.75	32.02
Role Function	62.50	38.73
Emotional Function	75.52	18.12
Cognitive Function	76.04	24.32
Social Function	55.21	32.04
Global Quality of Life	57.22	21.79

Symptom Scales (Higher scores = more symptomatic)		

Fatigue	41.67	25.82
Nausea	14.58	14.75
Pain	41.67	25.82
Dyspnea	22.92	26.44
Sleep	35.42	30.96
Appetite	33.33	32.20
Constipation	25.00	28.55
Diarrhea	14.58	17.78
Finances	29.17	26.87

Correlations between subscales are presented in Table 6 (additional file 1). The subscales of role function, cognitive function, global QL, fatigue and appetite loss were significantly related to seven other subscales each. Social functioning was associated with scores on six other subscales. Finances, diarrhea, and sleep were unassociated with any other subscales, and pain was associated only with dyspnea. All significant correlations were in the expected directions.

**Table 4:** SEIQoL Items

Patient Number	Item 1	Item 2	Item 3	Item 4	Item 5
1	Children	Spouse	Religion	Physical Fitness	Finances
2	Family	Exercise	Nature	Computer	Work
3	Spouse	Children	Friends	Activities	Father
4	Family	Friends	Dog	Gardening	Fun
5	Pain Control	Finances	Health	Energy	Activities
6	Travel	Health	Family and Friends	Spouse	Activities
7	Children	Family	Mobility	Hope	Work
8	Work	Family	Finances	Health	Activities
9	Family	Grandchildren	Friends	Travel with Spouse	Finances
11	Family	Friends	Active at Home	Work	Finances
12	Spouse	Friends	Family	Belief	Art
13	Activity	Grandchildren	Sewing	Gardening	Travel
14	Family	Faith	Positivity	Activities	Friends
15	Work	Recreation	Mobility	Family	Friends
16	Spouse	Children	Family	Faith	Exercise
17	Family	Spouse	Work	Family tree	Religion
Mean Level	70.9	68.4	64.6	56.9	62.5
SD	31.3	26.0	32.0	32.1	27.1
Mean Weight	0.25	0.23	0.19	0.20	0.16
SD	0.08	0.08	0.06	0.08	0.09

### Psychological Scores

Scores on the BDI, BSI and SHI are presented in Table 3. Fifteen of the 16 patients completed the questionnaires. Scores on the BDI averaged 11, in the moderate range of depressive symptomatology. On the BSI, scores ranged from a low of 0.17 on paranoid ideation, to a high of 0.91 on the obsessive-compulsive subscale. The overall global severity index was 0.55. These scores are higher than those of the general population, but quite a bit lower than those of psychiatric outpatients [21].

**Table 3:** Psychological Scores

	Mean	SD
BSI Somatization	0.74	0.56
BSI Obsessive Compulsive	0.91	0.63
BSI Interpersonal Sensitivity	0.38	0.36
BSI Depression	0.76	0.62
BSI Anxiety	0.50	0.40
BSI Hostility	0.21	0.27
BSI Paranoid Ideation	0.17	0.17
BSI Psychoticism	0.16	0.17
BSI Global Severity Index	0.55	0.36
Beck Depression Inventory Total Score	11.40	9.46
Spiritual Health Inventory Total Score	118.33	16.02

Patients scored an average of 118 on the SHI. The most highly endorsed items on the scale of 1–5, where 1 corresponds with the heading "not at all", and 5 with "very much", were the following: "I believe other people accept me even with my faults" (4.5); "I actively participate in decisions concerning my health care" (4.5); "I believe my nurses and doctors care about me" (4.3); "My life has a purpose" (4.2); "I feel accepted and forgiven despite some past actions" (4.0). The lowest scores were on the following items: "I wonder if God is angry with me" (1.1); "I feel angry with others" (1.3); "I feel a need to be forgiven for some of my thoughts and feelings" (1.7); "I worry about life after death" (1.9); "I feel angry with myself" (1.9); and "I feel out of touch with my own feelings and with others" (1.9).

### SEIQoL

All 16 patients completed the SEIQoL. The average time taken was 13.5 minutes, range 5–30 minutes. Areas identified by patients as the most important in determining their overall quality of life are presented in Table 4 by patient, along with average levels and weights associated with each cue by order of identification. As can bee seen, most patients identified family, children, or spouse as the single most important factor in determining their current quality of life. The average level of each of the five cues ranged from 57–71 on a scale of 0–100, where 100 was the best possible state for that cue. The weights assigned to the cues varied from 16% to 25%, a fairly narrow range, with those cues identified earlier in the process generally being assigned higher importance. The overall index scores, which take into account both the level of the cue and its weight, were an average of 69, SD 20.5 and ranged from 27–100. The frequency of nomination of different cues as any of the five domains is presented in Table 5. All but one patient mentioned some family relationship as one of the five domains, while some patients nominated several different specific family relationships. This was followed by the general ability to participate in chosen activities (e.g. exercise, recreation, travel, gardening, sewing). Seventy-five percent of the patients nominated some type of activity in their top five. The next most frequent category was friends, endorsed by 44% of the patients. This was followed equally by health (mobility, fitness, energy), faith (religion, belief, hope), and work, with 38% of the patients nominating each category. Finances were nominated by 31% of the patients, followed by several items that were mentioned by only one person each and warranted separate categories.

**Table 5:** Frequency of Cue Nomination

Cue	N (out of 16)	%
Family (Children, Spouse, Grandchildren, Parent, Family Tree)	15	93.8
Activities (exercise, gardening, sewing, recreation, travel)	12	75
Friends	7	43.8
Health (mobility, physical fitness, energy)	6	37.5
Faith (religion, belief, hope)	6	37.5
Work	6	37.5
Finances	5	31.3
Pet	1	6.3
Computer	1	6.3
Pain Control	1	6.3
Art	1	6.3
Fun	1	6.3
Positivity	1	6.3
Nature	1	6.3

The internal validity of each of the cues was assessed by performing regressions of the combination of each cue level and its weight onto the total index scores. The resulting R^2 ^values ranged from .19–.76, median .47, mean .50. The highest R^2 ^value was for the first cue generated, and the lowest value was associated with the fourth cue. This is much lower than in previous reports [16,17] and may constitute reason to pause before attributing high levels of credence to the validity of all of the cues in influencing overall QL.

**Figure 1:**
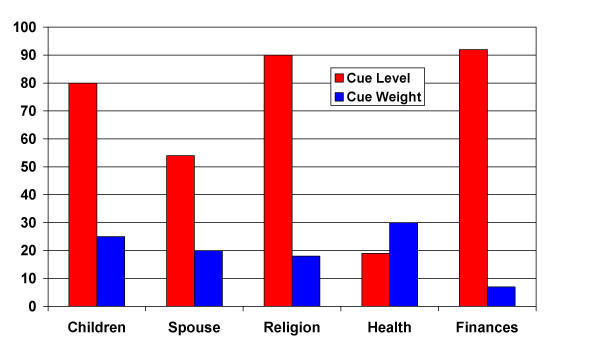
Patient #1: Cues, Levels and Weights

**Figure 2:**
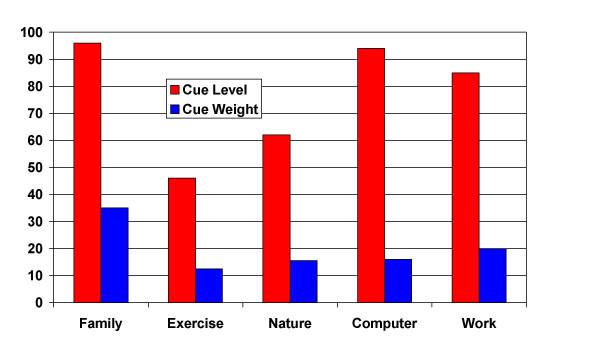
Patient #2: Cues, Levels and Weights

Two examples of cues, cue levels and cue weights are illustrated in Figures 1 and 2. Figure 1 illustrates the responses of patient #1, a 45 year old woman with mucoepidermoid cancer of her head and neck region who died four weeks following the interview. The most important areas to her were health, followed by children, spouse, religion and finally finances. This profile is unusual for this group in that most patients, if they nominated health as a cue at all, did so later in the process. She indicated that the areas that were going the best were finances and religion, followed by children, spouse and health. This combination of things not rated as going very well in some important areas resulted in an index score of 59 on the SEIQoL This is quite a bit higher than her global QL score on the EORTC of just 25. Another example is patient #2 (figure 2), a 50 year-old male with head and neck cancer who, as of this writing, has been alive for 709 days following the interview. For him, things were going well in the areas most important to him; family, work and computers. This resulted in an index score of 81, consistent with his global QL score on the EORTC of 75. These examples illustrate that the SEIQoL scores are related to overall QL scores, and suggest that they may be related to health status as well.

### Correlations between measures

Correlations between the SEIQoL index and scores on the other measures are presented in Table 6 (see additional file 1 – Carlson Table 6.doc). The index score was significantly positively correlated with the global QL score (r = .53, p < .05), and negatively associated with the symptoms of nausea (r = -.58, p < .05), pain (r = -.53, p < .05) and appetite loss (r = -.59, p < .05) on the EORTC QLQ C-30. Scores on the BDI were positively associated with appetite loss (r = 0.56, p < .05) and fatigue (r = 0.56, p < .05), not surprising since these are both symptoms of depression assessed by the BDI. Depression scores were also negatively related to social functioning (r = -.79, p < .01) and global quality of life (r = -.69, p < .01), indicating that people who endorsed more depressive symptoms also tended to report lower social functioning and lower overall QL. Higher scores on the Global Severity Index of the BSI were associated with worse emotional (r = -.55, p < .05), cognitive (r = -.58, p < .05) and social (r = -.69, p < .01) functioning, as well as worse global QL (r = -.70, p < .01) and more sleep disturbance on the EORTC. Higher scores on the spiritual health inventory were associated with lower scores on the global severity index of the GSI (r = -.54, p < .05) and less nausea (r = -.63, p < .05).

Significant correlations between days to death and psychological scores were found on two instruments in the nine patients who had passed away at the time of writing. The BDI total score was negatively correlated (r = -.75, p < .05), and the EORTC global QL score was positively correlated (r = .77, p < .05) with time to death. This indicates an association between higher levels of depression and lower global QL at the time of the interview, and fewer days to death.

## Discussion

This study is the first to use the SEIQoL-DW instrument to assess individualized QL in a population of patients with advanced cancer participating in a Phase I clinical trial. Of note is that the instrument was easy to use in this population and acceptable even to those patients who were quite ill. Replication of the wide range of individual differences in the cues chosen and the weights associated with each cue was seen in this group. The areas of QL that were nominated by patients as the most important factors in the determination of their overall QL were primarily family relationships, the ability to participate in pleasurable activities, and friendships. Only 38% of this sample mentioned health or health-related domains such as mobility, fitness and energy as one of the five cues. This is in contrast to other samples of cancer patients where over 70% of patients nominated health as an important domain. For example, health was nominated by 73% of cancer patients in phase I trials (not necessarily advanced cancer) [18], 87% of men with early stage prostate cancer [16], and 70% of patients with advanced incurable cancer (these patients were not on trials) [17]. The commonality between these studies is that family was consistently nominated as the most frequent domain. In terms of the index scores, our average of 69 was higher than that of the group of advance incurable cancer patients (mean = 58) and the patients participating in Phase I trials (mean = 61). It was comparable to the men with early stage prostate cancer (mean = 71), but the relatively small range of mean scores among these studies is notable. Construct validity is supported in that those patients whom one might expect to have higher QL (i.e. less ill patients), indeed reported higher QL. That the scores in the current sample were more comparable to the early stage prostate cancer patients than the incurable cancer patients may speak to the hope patients were feeling regarding the potential of the reovirus treatment.

In terms of the index scores for other illness populations, patients prior to hip arthroplasty scored 59, after the surgery scores increased to 69 [27], on par with the cancer patients in this study (mean 69). Another study of hip replacement patients found scores of 62 prior to surgery, with improvements to 71 following surgery [28], a similar improvement pre- to post-surgery. Severely disabled multiple sclerosis patients scored 61 [29], patients with ALS had a higher mean index of 76 [30], and cardiac patients scored a high index of 82 after myocardial infarction or coronary artery bypass graft surgery and prior to beginning cardiac rehab [31];. Interestingly, only 24% of the ALS patients nominated health (disease progression) as a cue. Thus, although the areas of importance varied by individual in all these studies, the resultant index scores seem to demonstrate some consistency across similar populations.

Another indication of the construct validity of the SEIQoL-DW is its high correlation to global QL scores on the EORTC QLQ C-30. This speaks to the validity of the self-generated items, as the sum of the products of their importance and current status was associated with the overall global assessment of QL on standardized domains. Predictably, our patients had a global QL on the EORTC of 57, much lower than the 77 reported in a large normative community sample [32]. Scores on all five functional scales and symptom scores were all also worse in this population, not surprising considering the extent of their disease status. However, they did have the same global QL scores compared to a large sample of patients with advanced malignancy from 12 institutions in 10 countries (both 57), but scored higher on most of the other functional scales than this group (Physical function: 64 vs. 60; Role function: 63 vs. 50; Emotional function: 76 vs. 50; Cognitive function: 76 vs. 63; Social Function: 55 vs. 50) [33]. The greatest differences in favor of the patients in this trial were seen on emotional, cognitive and role function.

Interestingly, lower global QL scores on the EORTC and higher depression scores on the BDI were associated with a shorter time to death in those patients who had already passed away, an association that has been reported in other studies [33-35]. In fact, in an international sample of 411 patients with advanced malignancy, similar to the current group, the single-item global QL scale remained independently prognostic of death in a proportional hazards model stratified on diagnostic category, after allowing for performance status and age, and, among solid tumor patients, metastatic site [33].

Patients who were in terminal care from a large sample from 12 oncology outpatients departments around the United States scored an average BSI Global severity index score of .93. Those who were under symptom control scored .80, whereas lower scores were seen for patients in active therapy (.59), adjuvant therapy (.60) or no current therapy (.65)[36]. Our sample mean of .55 is quite low in comparison and most comparable to patients in active therapy. An even larger sample of over 4000 patients of all disease sites and stages of illness from Johns Hopkins Oncology Centre reported an average global severity index very similar to our patients, at .54 [37]. This seems to indicate that our sample is reporting less psychopathology than would be expected of patients in similar disease states, but comparable levels to cancer patients in general. They also scored an average of 11 on the BDI, which indicates mild depressive symptomatology.

The spirituality scores of this group average 118. This is very similar to a group of lung cancer patients who had a mean of 120 [20]. Analysis of the individual items showed that these patients endorsed feeling well supported by the medical team and quite peaceful and accepting of themselves, and well accepted by others. They reported not feeling angry at themselves or others, or worried about life after death. The spirituality scores were correlated with the global severity index of the BSI, indicating that those who felt more spiritually at ease also endorsed fewer symptoms of psychopathology.

In summary, these patients in a Phase I trial of a promising novel therapeutic were easily able to complete the SEIQoL-DW interview as well as a battery of other psychological questionnaires. They reported feeling excited and hopeful about the trial, with about two-thirds hoping for disease regression, and another third optimistically hoping for a cure. However, most acknowledged that although they hoped for the best they were realistic in their expectations. The individuality of QL as defined by each person was reinforced in this group, as many different cues were nominated as important and variable weights were assigned to the same cues. Consistent with reports from other seriously ill groups, health status received less focus than other aspects of life, primarily family relationships and activities. Overall QL on standardized measures and psychological status was generally better than other seriously ill patient groups, but comparable to cancer patients in general. QL and depression scores were related to time until death in those patients who had passed away.

## Authors' contributions

LC wrote the research proposal and ethics applications, conducted the interviews with the patients, designed the database, conducted the statistical analysis, and wrote the first draft of the paper. BB is the department head for Psychosocial Resources, and in conjunction with LC conceived of and designed the study. He also made the linkages with medical oncology to facilitate the study. DM is the oncologist and principal investigator of the Phase I Reovirus trial, and recruited all the patients. All authors reviewed and edited the final manuscript.

## Supplementary Material

Additional File 1 Table 6: Correlations between scores on the SEIQoL, BDI, BSI, SHI and EORTC QLQ C-30 Click here for file
