# Supplementary material for: Individualized quality of life, standardized quality of life, and distress in patients undergoing a phase I trial of the novel therapeutic Reolysin (reovirus)
Source: Health Qual Life Outcomes. 2005 Jan 27;3:7. doi: 10.1186/1477-7525-3-7 (PMC548292; doi:10.1186/1477-7525-3-7)
Supplement: Additional File 1 — Table 6: Correlations between scores on the SEIQoL, BDI, BSI, SHI and EORTC QLQ C-30 [file 1477-7525-3-7-S1.doc]

Table 6: Correlations between scores on the SEIQoL, BDI, BSI, SHI and EORTC QLQ C-30

|  | 1 | 2 | 3 | 4 | 5 | 6 | 7 | 8 | 9 | 10 | 11 | 12 | 13 | 14 | 15 | 16 | 17 | 18 |
| --- | --- | --- | --- | --- | --- | --- | --- | --- | --- | --- | --- | --- | --- | --- | --- | --- | --- | --- |
| 1. SEQoL Index |  |  |  |  |  |  |  |  |  |  |  |  |  |  |  |  |  |  |
| 2. BDI | -.13 |  |  |  |  |  |  |  |  |  |  |  |  |  |  |  |  |  |
| 3. BSI GSI | -.11 | .70 |  |  |  |  |  |  |  |  |  |  |  |  |  |  |  |  |
| 4. SHI | .33 | -.45 | -.54* |  |  |  |  |  |  |  |  |  |  |  |  |  |  |  |
| 5. Physical | .44 | -.65 | -.43 | .42 |  |  |  |  |  |  |  |  |  |  |  |  |  |  |
| 6. Role | .24 | -.48 | -.45 | .27 | .82** |  |  |  |  |  |  |  |  |  |  |  |  |  |
| 7. Emotional | -.18 | -.38 | -.55* | .43 | .36 | .58* |  |  |  |  |  |  |  |  |  |  |  |  |
| 8. Cognitive | -.15 | -.71 | -.58* | .37 | .49 | .52* | .56* |  |  |  |  |  |  |  |  |  |  |  |
| 9. Social | .37 | -.79** | -.69** | .42 | .67** | .75** | .43 | .69** |  |  |  |  |  |  |  |  |  |  |
| 10. Global QL | .53* | -.69** | -.70** | .44 | .73** | .59* | .40 | .53* | .75** |  |  |  |  |  |  |  |  |  |
| 11. Fatigue | -.21 | .56* | .59* | -.25 | -.74** | -.82** | -.45 | -.72** | -.76** | -.71** |  |  |  |  |  |  |  |  |
| 12. Nausea | -.58* | .34 | .38 | -.63* | -.55* | -.44 | -.34 | .06 | -.37 | -.58* | .21 |  |  |  |  |  |  |  |
| 13. Pain | -.53* | .08 | .33 | -.03 | -.39 | -.39 | -.27 | -.22 | -.37 | -.60* | .46 | .10 |  |  |  |  |  |  |
| 14. Dyspnea | .03 | .41 | .32 | -.42 | -.42 | -.30 | -.18 | -.70** | -.37 | -.33 | .61* | -.15 | .14 |  |  |  |  |  |
| 15. Sleep | .05 | .27 | .67** | -.28 | .04 | -.02 | -.33 | -.13 | -.20 | -.28 | .16 | .25 | .16 | .12 |  |  |  |  |
| 16. Appetite | -.59* | .56* | .48 | -.44 | -.73** | -.62** | -.13 | -.57* | -.79** | -.80** | .74** | .39 | .45 | .52* | .00 |  |  |  |
| 17.Constipation | -.38 | .06 | -.15 | .50 | -.26 | -.10 | .40 | .17 | -.03 | -.26 | .24 | .04 | .30 | -.22 | -.15 | .16 |  |  |
| 18. Diarrhea | .17 | -.01 | -.38 | .13 | -.03 | .04 | -.09 | -.17 | .06 | .05 | -.07 | -.31 | .13 | .20 | -.34 | -.14 | -.04 |  |
| 19. Finances | -.30 | -.05 | -.21 | -.04 | .07 | .16 | .04 | .23 | .07 | -06 | -.30 | .07 | .05 | -.27 | -.26 | -.26 | .05 | .30 |

* p<.05, ** p<.01
